# Expression of miR-34 is lost in colon cancer which can be re-expressed by a novel agent CDF

**DOI:** 10.1186/1756-8722-5-58

**Published:** 2012-09-19

**Authors:** Sanchita Roy, Edi Levi, Adhip PN Majumdar, Fazlul H Sarkar

**Affiliations:** 1 Department of Veterans Affairs Medical Center, Wayne State University, Detroit, MI, 48201, USA; 2 Departments of Internal Medicine, Wayne State University, Detroit, MI, 48201, USA; 3 Departments of Pathology, Wayne State University, Detroit, MI, 48201, USA; 4 Departments of Oncology, Karmanos Cancer Institute, Wayne State University, Detroit, MI, 48201, USA; 5 Departments of Pathology and Oncology, Karmanos Cancer Institute, Wayne State University School of Medicine, 740 HWCRC, 4100 JohnR Street, Detroit, MI, 48201, USA

**Keywords:** MiR34a, MiR-34c, Colon cancer, CDF, Methylation

## Abstract

**Background:**

Colorectal Cancer (CRC) is one of the leading causes of death worldwide. Numerous cellular events, including deregulated expression of microRNAs (miRNAs), specifically the family of miR-34 consisting of miR-34a, b and c, is known to regulate the processes of growth and metastasis.

**Methods:**

We evaluated the expression of miR-34 in formalin-fixed paraffin-embedded (FFPE) human colon cancer tissue specimens compared to normal colonic mucosa. Moreover, we also assessed the expression of miR-34 in colon cancer cell lines treated with our newly developed synthetic analogue of curcumin referred as difluorinated curcumin (CDF) compared to well known inhibitor of methyl transferase.

**Results:**

We found that the expression of miR-34a and miR-34c was down-regulated in colon cancer specimens compared to normal colonic mucosa and the loss of expression was also consistent with data from colon cancer cell lines. This down-regulation was attributed to promoter hypermethylation, because we found that the treatment of colon cancer cells with 5-aza-2´-deoxycytidine, a methyltransferase inhibitor, markedly induced the levels of miR-34a and miR-34c expression. Likewise, CDF was very effective in the re-expression of miR-34a and miR-34c, which was consistent with inhibition of cell growth of both chemo-sensitive and chemo-resistant colon cancer cells. The re-expression of miR-34 led to a marked reduction in the expression of its target gene, Notch-1.

**Conclusion:**

The loss of expression of miR-34 in colon cancer is in part due to promoter hypermethylation of miR-34, which can be re-expressed with our novel agent CDF, suggesting that CDF could be a novel demethylating agent for restoring the expression of miR-34 family, and thus CDF could become a newer therapeutic agent for the treatment of colon cancer.

## Introduction

Colorectal cancer (CRC) is the third most common cancer in women and the fourth in men [1]. Little over 1.2 million cases are diagnosed each year globally with about 600,000 deaths. The primary cause of colon cancer induced death is due to metastasis to the liver [2]. Nearly, 50 % of the patients diagnosed with colorectal cancer show tumor recurrence, which is assumed to be due to the presence of chemotherapy-resistant cancer stem cells (CSCs) [3]. Therefore, newer treatment strategies are urgently needed for reducing the rate of recurrence and thereby improving the overall survival of patients diagnosed with colorectal cancer. We have focused our investigation to finding ways to restore the expression of specific microRNAs (miRNAs) that are down-regulated in colorectal cancer and are involved in the progression of this malignancy.

The miRNAs are a class of endogenous small non-coding RNAs that control gene expression through binding to the seed sequence at the 3´-UTR of target mRNAs, resulting in translational repression or mRNA degradation [4]. It has been predicted that over 30 % of the human protein coding genes are post-transcriptionally regulated by this mechanism [5]. miRNAs have also been shown to regulate numerous processes of carcinogenesis, including the growth and maintenance of cancer stem-like cells (CSLCs) which are known to be resistant to chemotherapy and possess the limitless capacity to regenerate [6,7]. The CSLCs play critical roles in the development and progression of many malignancies, including colorectal cancer [8]. Family of miR-34 that includes 34a, b and c has been reported to inhibit CSLCs [9]. They are down-regulated in colorectal cancer [10] which may contribute to the progression of the disease as well as drug resistance[11]. Emerging evidence suggests that p53 acts as a transcription factor to increase the expression of the miR-34 family members which, in turn, modulate cell cycle progression, senescence and apoptosis, inhibition of invasion and migration [12,13]. Interestingly a positive feedback loop exists between p53 and miR-34a [14]. The p53-induced expression of miR-34a inhibits its target gene SIRT1, a histone deacetylase. Down-regulation of SIRT1 expression up-regulates p53 acetylation and the transcriptional activity of p53 [15]. Indeed, up-regulation of miR-34 has been shown to induce cell-cycle arrest, inhibition of invasion and migration and p53 induced apoptosis [16,17]. In view of this, it is tempting to speculate that p53-mediated processes of apoptosis in colon cancer cells could be affected by down-regulation of miR-34. However, little is known whether agent(s) that modulates colon CSLCs would also modulate the family of miR-34 in colon cancer cells or not.

In search of such agents, we tested the effects of our recently generated difluorinated curcumin (CDF), a novel analog of the dietary ingredient curcumin, with much greater bioavailability than the parent compound [18,19]. Recent data from our laboratory suggest that CDF´s anti-tumor activity is mediated by multiple mechanisms including regulation through miRNAs [20-23]. CDF also causes a marked inhibition of cellular growth and induces apoptosis in chemo-resistant (5-Fluorouracil and Oxaliplatin-resistant) colon cancer cells, and demonstrates a remarkable ability to disintegrate colonospheres [24] that are considered to be surrogate tumors [25]. Suffice to mention that the chemo-resistant colon cancer cells and colonospheres are highly enriched in CSLCs [25,26]. This relevant information prompted us to determine whether CDF could be utilized to modulate the family of miR-34, and if so, whether CDF-induced modulation of miR-34 could in part be attributed to epigenetic alterations, specifically the methylation status of the promoter of miR-34.

## Materials and methods

### Cell lines and culture condition

While the colon cancer cell line SW620 was maintained in RPMI and HCT116wt (p53 wt, K-ras mutant), HCT116P53−/−, HCT116 CR were maintained in DMEM (Invitrogen). All cell lines were supplemented with 10 % fetal bovine serum (FBS), 1 % antibiotic-antimycotic and maintained in 5 % CO2-humidified atmosphere at 37 °C. The medium was changed twice a week, and the cells were passaged using 0.05 % trypsin-EDTA (Invitrogen). The 5-Fluorouracil and Oxaliplatin- resistant HCT116 (HCT116 CR) cells were generated by us and maintained as described previously [27].

### Treatment of colon cancer cell lines

5-Aza-2'-deoxycytidine (SIGMA-Aldhrich) and CDF were dissolved in DMSO. HCT116wt, HCT116p53−/−, HCT116 CR cells were treated with 100 nM CDF alone; SW620 cells were treated either with 5 μM 5-Aza-2'-deoxycytidine (Aza-dC) or 100 nM CDF for 72 hours. Control cells received 0.05 % DMSO. Fresh Aza-dC, CDF and DMSO were added everyday along with a change of medium. After 72 hours, cells were subjected to DNA, RNA, and protein extraction.

### Colon cancer tissue specimens

Archival formalin-fixed paraffin-embedded tissues from normal colonic mucosa and colon tumors were obtained from the Pathology Service of the John D. Dingell VA Medical Center; Detroit through the Wayne State University IRB approved protocol to isolated RNA for assessing the expression of miR-34 family. No information of patients was retrieved.

### Real-time RT-PCR

To determine the miRNA-34 levels, RNA isolated from FFPE tissues using miRNeasy FFPE Kit (Qiagen) and from cultured cells using miREasy kit (Qiagen) was utilized. Twenty naogram of total RNA were reverse transcribed into cDNA using a Universal cDNA Synthesis Kit (Exiqon, Woburn, MA) according to the manufacturer's protocol. Real time PCR was performed using specific primers for miR-34a, miR34c (Exiqon) and SYBR® Green PCR Reagents (Applied Biosystems). The relative amount of miRNA was normalized to the expression of RNU1α (Exiqon).

### Methylation-specific PCR

Genomic DNA was isolated by using the Wizard Genomic DNA Purification Kit (Promega). Five hundred nanograms of genomic DNA were treated with bisulfite using the EZ DNA Methylation-Gold Kit (Zymo Research). The modified DNA was eluted in a final volume of 10 μl, and 100 ng was used for the methylation-specific PCR. Methylation specific PCR for miR34a promoter was carried out using ZymoTaq DNA Polymerase (Zymo Research) according to the PCR condition described by Lodygin [28]. Quantification of methylated vs unmethylated promoter was done by real time PCR as described previously [29].

### Western blot analysis

Western blot analysis was performed according to standard protocol [22]. Briefly, the cells were lysed in lysis buffer and the protein concentration was determined by the Bio-Rad Protein Assay kit (Bio-Rad). The proteins were separated by SDS-PAGE and transferred to polyvinylidene difluoride (PVDF) membranes (Millipore). The membranes were blocked with BSA at room temperature for 1 h, subsequently incubated overnight at 4^0^C with primary antibodies to Notch 1 (Santa Cruz; sc-6014). The membranes were then washed and incubated with appropriate secondary antibodies. The protein bands were visualized by ECL prime western blotting detection reagent (GE Healthcare Biosciences). The membranes were stripped as needed for further analysis.

### Statistical analysis

Results are presented as the mean ± SD. For cell culture data comparisons of the continuous variables between two independent groups were calculated using two-tailed student's t test. For unpaired patient samples, comparisons of the continuous variables between two independent groups were made using the Mann Whitney test. The p value of <0.05 was considered to be statistically significant.

## Results

### miR-34a and miR-34c are down-regulated in colon cancer

**Figure 1:**
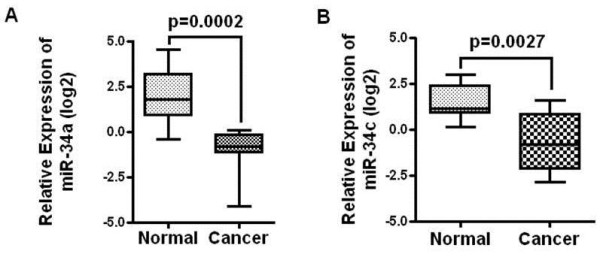
** miR-34a and miR34c are downregulated in colon cancer.** Quantitative real-time RT PCR was performed with RNA isolated from formalin fixed paraffin embedded (FFPE) normal and colon cancer tissues (n = 10 for each group) to determine the expression of miR34a (**A**) and miR34c (**B**).

The levels of miR-34a and miR-34c in histologically normal and colon cancer tissues, as determined by quantitative real time RT-PCR revealed that both miR-34a and miR-34c were significantly down-regulated in colon cancer (Figure 1A, B). This observation is similar to what has been previously reported by others [10]. 

### CDF up-regulates miR-34a and miR-34c in different colon cancer cell lines

Members of the miR-34 family act as a tumor suppressor, hence their reduction or loss in colonic mucosa leads to malignancy as reported in other cancers [30]. So far, no studies have been performed to determine whether of miR-34 could be expressed in colon cancer by any novel agent(s). Our primary objective was, therefore to determine whether, CDF would modulate miR-34 expression in colon cancer cells.

To investigate whether the effect of CDF on miR-34 expression is p53 dependent, we extended our study using HCT116p53−/− and SW620 (p53 mutant, where G > A mutation in codon 273 of the p53 gene results in an Arg > His substitution) cell lines. Additionally, we used HCT116 CR cells to examine how CDF acts for miR34 expression in drug resistance.

**Figure 2:**
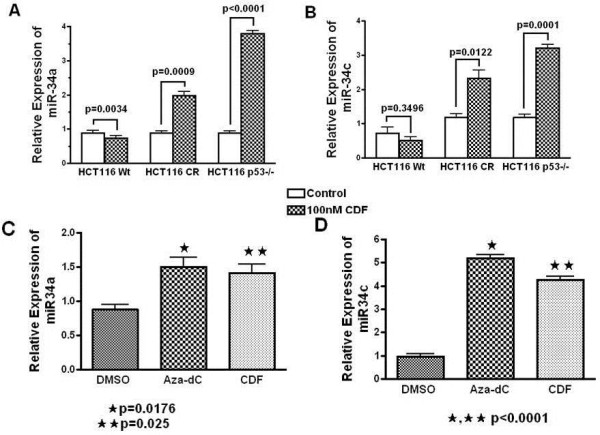
** CDF upregulates the expression of miR-34a and miR-34c in different colon cancer cell lines.** (**A**) The miR-34a and (**B**) miR-34c expression was increased significantly in HCT116CR, HCT116p53−/− cells following treatment with CDF. (**C**) The miR-34a and (**D**) miR-34c expression was also up-regulated significantly in SW620 cells treated with either CDF or Aza-dC for 72 h. ( DMSO, Aza-dC, CDF in the figure are DMSO, 5 μM of 5-Aza-2'- deoxycytidine, 100nM of CDF treated cells. * P, ** P denote the level of significance in Aza-dC or CDF treated cells, compared to DMSO-treated controls). The quantitative values were expressed as means ± SD of triplicate measurements, and are representative of two separate experiments.

Our current data demonstrated that CDF significantly induced the expression of both miR-34a and miR-34c in HCT116CR, HCT116p53−/− and SW620 that were either in chemo-resistant or p53-defficient (Figure 2). Interestingly, re-expression of miR34a and miR34c levels was found to be greater in HCT116p53−/− cells indicating that CDF may act independent of p53 status (Figure 2A, 2B). Although the reason is unclear, why CDF could not induce miR-34 in HCT116Wt cell, one possibility could be the over growth making CDF less available to induce miR-34.

### CDF demethylates miR-34a promoter to up-regulate the expression of miR-34a

Silencing of miR-34 expression due to its promoter hypermethylation of the CpG site has been documented in colon cancer [28,31], suggesting that the use of demethylating agents like azacitidine (Aza-dC) and decitabine could be useful for the treatment of solid tumors [32] although these agents show unacceptable side effects. Assuming that CDF may act as a demethylating agent, we compared the effects of CDF with Aza-dc on the expression of miR-34a and miR-34c in colon cancer SW620 cells. As shown in Figure 2C and 2D, both CDF (experimental agent) and Aza-dC (control agent), led to increased expression of miR-34a and miR-34c in SW620 cells. We further extended our study for CpG methylation analysis of the miR-34a promoter only, since its expression was found to be relatively higher than miR-34b, miR-34c in all human tissues except lung [33].

**Figure 3:**
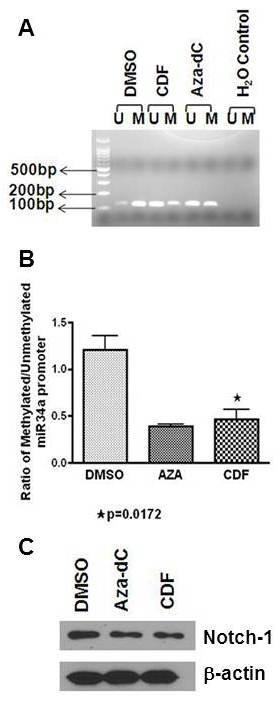
** Reversion of miR-34a silencing in SW620.** (**A**) Agarose gel electrophoresis and (**B**) qRT PCR quantitation showing miR-34a promoter methylation (M: methylated and U: unmethylated products); (**C**) Re-expression of miR-34a by Aza-dC and CDF treatment decreased the expression of its downstream target Notch-1.

In the current investigation, we used SW620 cells instead of HCT116 cells because HCT116 cells has been reported to be negative for methylation specific PCR of miR-34a promoter where the induction of miR-34a was not associated with its promoter methylation but possibly through alternate mechanism [28]. SW620 cell line was found to be positive for CpG methylation of miR-34a promoter. The CpG methylation of miR-34a promoter was found to be reverted following 72 h exposure to CDF (Figure 3A, B). Interestingly, CDF treatment not only led to an increased expression of miR-34a but also decreased the expression of its downstream target Notch-1 (Figure 3C) in SW620 cells, suggesting that induction of miR-34a is directly responsible for its functional activity**.**

## Discussion

Colorectal cancer, an age-related disease whose incidence increases sharply with advancing age, is a multistage process involving both genetic and epigenetic alterations[34]. More recently, it has come to light that microRNAs are capable of exerting pleiotropic effects on cancer cells by post-transcriptional regulations of numerous genes[4]. Hence, it is not surprising that miRs have been shown to be dysregulated in various human malignancies including colorectal cancer [35].

The family of miR-34, that includes miR-34a, b and c, has been known to regulate several cellular events, including cell cycle, cell migration and apoptosis [16,17]. Our current observation on colorectal cancer tissues and those reported by others show that miR-34 is downregulated in colorectal cancer, suggesting that downregulation of this microRNA may partly contribute to the unregulated cellular growth and drug resistance that occurs in colorectal cancer.

It is essential to develop the strategy for restoring the expression of miRs specifically the family of miR-34 which are dysregulated in cancer. Our current observation that CDF induces the expression of miR-34a and miR-34c in chemo resistant and p53 defficient colon cancer cells, which suggests that CDF is effective in re-expressing miR-34 and could be a potential therapeutic agent for colorectal cancer. Chemotherapy resistance is a major concern of colon cancer treatment. Though FOLFOX (combination of 5-FU, Leucovorin and Oxaliplatin) is the mainstay of colon cancer treatment, but it failed to eradicate all tumor cells, resulting in tumor recurrence [3]. To gain further insight into the drug-resistance in colon cancer, we generated chemo-resistant human colon cancer cell line HCT116 CR [27] which was used earlier to assess the effects of CDF on the growth, apoptosis and colon CSLCs elimination [24]. CDF inhibited the growth of chemo-resistant colon cancer cells and induced disintegration of colonospheres [24]. The fact that CDF restores expression of miR-34a and 34c in chemo-resistant colon cancer cells, highly enriched in CSLCs suggests that CDF could be effective in arresting the growth of colon CSLCs that are known to be resistant to conventional chemotherapy [36].

Although the precise mechanism(s) by which CDF induces miR-34a and miR-34c has not been fully elucidated, our current data suggest that demethylation of the respective promoter of miR-34a and miR-34c by CDF could be one possibility. The expression of miR-34a and miR-34b/c in different cancers has earlier been shown to be silenced by CpG methylation in the promoter region in different cancer [31]. Therefore, demthylation is likely to enhance the expression of miR-34. This inference is supported by the observation that in colon cancer SW620 cells, the level of methylated promoter of miR-34a was decreased in response to CDF treatment, and that this reduction was accompanied by concomitant reduction in Notch-1 expression, one of the targets of miR-34a which is consistent with our previous finding on Notch-1 expression after CDF treatment and after miR34a induction mediated by other demethylating agents [23,29]. However to date there is no direct proof that CDF is a direct inhibitor of DNMT or not, which requires further in-depth investigation in the future.

In conclusion, our findings demonstrate that the expression of miR-34a and miR-34c is greatly reduced in colon cancer. CDF, a novel analog of curcumin, which has earlier been shown to induce apoptosis of chemo-resistant colon cancer cells, and it can re-express miR-34a and 34c. The latter could be partly attributed to demethylation of the respective promoter of the miR-34a and 34c, which require further mechanistic in-depth investigation.

## Competing interests

None of the authors have any competing interest and there is no conflict.

## Author's contributions

Drs. SFH and MAPN designed experiments. Dr LE provided FFPE blocks of human colon cancer and normal colonic tissue samples. RS carried out all experiments, analyzed data and prepared the manuscript. Drs. MAPN and SFH served as the principle investigators, and were critically evaluated the work and undertook overall supervision of the preparation of the final manuscript.
